# An Account of the Singular Effects of Music on a Patient

**Published:** 1790

**Authors:** James Lucas

**Affiliations:** one of the Surgeons to the General Infirmary at Leeds


II. *An Account of the ſingular Effects of Muſic on a Patient. By Mr.* James Lucas, *one of the Surgeons to the General Infirmary at Leeds.*

J. E., aged twenty years, the ſon of a reſpectable farmer at Haſewood, was ſeized with a ſlow fever in March, 1759, by which, in eight weeks, he was reduced to a very weak ſtate. He was a ſtranger to any intemperance; and although of a grave diſpoſition, there was no reaſon whatever to ſuſpect inſanity.

On the 29th of May a company of young perſons paſſed by the houſe, carrying oaken boughs, and playing upon a fiddle: he no ſooner heard the muſic than he ſtarted up in bed, ſeemed tranſported with joy, and cried aloud, "Dance! dance!"

The young man had played upon the flute, but had never been accuſtomed to dance.

For

For two or three days he continued, almoſt inceſſantly, calling out, "Dance! dance! For "God's ſake let me dance;" though his father took uncommon pains to convince him of the impropriety and danger of fatiguing himſelf when in ſo weak a condition.

On the arrival of a muſician he exerted himſelf in putting on his cloaths, and immediately, upon hearing a ſtring of the violin touched, he ſtarted back, and for a while ſtood motionleſs; but, upon a tune being played, he danced with great agility, though in a ſtrange, frightful, and ſeemingly involuntary manner. The muſic would often cauſe him at firſt to move a hand; then a foot; a nodding of the head would ſometimes follow; and he would ſuddenly ſtart from his chair, make the moſt ridiculous and antic diſtortions with the muſcles of his face, and ſhake his limbs as if he was by deſign acting the part of a ſcaramouch or merry Andrew. After he had fallen upon the floor, the continuing of the muſic would repeatedly rouſe him, until fatigue prevented him from being affected by the ſound.

To this very ſtrange inclination he was ſubject daily for about three weeks: the affection ſometimes remained from ten to twenty minutes, nutes, at other times for two or three hours together.

His furious and menacing geftures caufed ftrangers to be much alarmed with him. He had a great averfion to a gloomy countenance, but feemed to be much pleafed with a cheerful one. Although his motions were too rapid to be voluntary, yet they appeared to keep time with the mufic.

Slow tunes, or even changing the tune, provoked him much, unlefs it was to a more lively one: if the change was made even the following day, he was immediately fenfible of it.

He would, for fome hours together, have a fecret wifh for mufic, though he did not difcover it, until it was conjectured by his filence and anxiety that he wanted it.

As he was once dancing with great alertnefs, it happened that a ftring of the fiddle broke; and although the mufician continued to play upon three ftrings, the young man ftood motionlefs, and was for a long time much out of humour, faying, that he was unable to defcribe the difagreeable fenfations produced in him by this accident.

The fatigue of dancing made him perfpire profufely, and he was frequently obliged to go to to bed immediately; yet he was fo much relieved by it, that he flept better, and in a few days after he began this exercife he was able to walk near half a mile to church. Upon obferving that his fpirits were low in the evening, the fiddler was mentioned; when he acknowledged that he had been endeavouring to conceal his defire to have him, becaufe it was Sunday.

During thefe uncommon attacks, a tune called Tarantula was played to him, which caufed him to move after other tunes had failed; but this was fuppofed to depend merely on its being a very lively air.

When the fit was gone off, the actions entirely fubfided, and he expreffed great pleafure at the relief which it had afforded him.

Such fevere exercife frequently produced a ftiffnefs and itching in his limbs: to remove this uneafinefs he would fometimes prick them with holly until they bled.

Inftead of the periodical inclination for mufic, he, by degrees, became feized with convulfive fits, during which his hands were clinched, his limbs ftiff and immoveable, his eyes rolled, his countenance was wild; he kicked off the bed cloaths; would not fuffer any one to fpeak to him, or fo much as to look at him; him; he became infenfible, fpeechlefs, and had a locked jaw, which once continued fo as to prevent him taking any nourifhment for at leaft twenty-four hours.

The convulfions fometimes ceafed in a quarter of an hour, at others continued for many hours; at firft they were flight, and foon over, but were afterwards more violent, and of longer duration. Upon his recovery, he remarked, that he did not fuffer fo much, during a fit, as his friends feemed to apprehend. His fpafmodic fymptoms gradually abated, but were fucceeded by a hectic fever, of which he died the latter end of December.

Dr. Dealtry, of York, and feveral other medical gentlemen were confulted in this uncommon cafe. The diforder was faid by fome to be St. Vitus's * dance; but, befides the effects of mufic, the motions fubfiding, and only returning at the time of a fit, rendered the complaint materially different. It did not appear that the patient had been bitten by any infect, or had read any account of the tarantula before the 29th of May; nor is it any wife probable

* For an account of a peculiar diforder, accompanied with laughing and dancing, fee Heifter's Medical Cafes, (Obf. 414) 4to. London, 1755.

 that that he was one of those impostors described as tarantulists *.

